# The incidence of necrotic enteritis in turkeys is associated with farm, season and faecal *Eimeria* oocyst counts

**DOI:** 10.1186/s12917-021-03003-8

**Published:** 2021-09-04

**Authors:** Magne Kaldhusdal, Eystein Skjerve, Magne Kjerulf Hansen, Inger Sofie Hamnes, Bruce David, Skjalg Arne Hanssen, Atle Løvland

**Affiliations:** 1 Department of Food Safety and Animal health, Norwegian Veterinary Institute, P.O.B. 750, Sentrum, 0106 Oslo, Norway; 2 Department of Production Animal Sciences, Faculty of Veterinary Medicine, Norwegian University of Life Sciences, P.O.B. 369, Sentrum, 0102 Oslo, Norway; 3 Norwegian Meat and Poultry Research Centre Animalia, P.O.B. 396, Økern, 0513 Oslo, Norway; 4 Nortura SA, P.O.B. 360, Økern, 0513 Oslo, Norway

**Keywords:** Epidemiology, Age, OPG, Feed mill, Grow-out size, Production performance

## Abstract

**Background:**

Specific studies on the epidemiology of necrotic enteritis in turkeys are absent in the literature. Necrotic enteritis is common in turkeys and a leading cause of use of therapeutic antibiotics. This study describes the incidence of necrotic enteritis in turkey farms, and the association between incidence and bird age, season, faecal oocyst counts, grow-out size and feed mill.

**Results:**

Necrotic enteritis was diagnosed post mortem in 20.2 % of 545 grow-outs of commercial female and male B.U.T. 10 turkeys started during the years 2010–2016. 80 % of all cases occurred at four to seven weeks of age. Median (minimum-maximum) age at disease detection was 37 (18–115) days. Turkey age at detection was influenced by season, and varied from 33 days among grow-outs hatched in February to 42 days among those hatched in July-August. The incidence also varied with season, showing peak occurrence among grow-outs hatched during February-March and the lowest incidence in turkeys hatched in July-August. 59 % of all cases were detected in 25 % of the farms. The incidence per farm varied from below 4 to 59 %. A multilevel mixed-effects logistic regression model indicated clear impacts of farm and season on incidence, and border-line impacts of grow-out size and feed mill. Grow-outs diagnosed with necrotic enteritis had higher counts of faecal *Eimeria* oocysts than grow-outs without a diagnosis. This difference was particularly clear during the high-risk period at five to seven weeks of age. Necrotic enteritis was the cause of treatment with therapeutic antibiotics in 88.2 % of all cases of treatment.

**Conclusions:**

Our data indicate that necrotic enteritis incidence in turkeys can be substantially influenced by risk factors at farm level. The incidence showed two seasonal peaks; a moderate peak in turkeys hatched in October/November and a marked peak in turkeys hatched during February/March. Mitigation measures at the farm may therefore be of particular importance during these months in farms located in the Northern temperate zone. Measures which effectively reduce counts of faecal *Eimeria* oocyst are likely to be among the more promising actions to take both at the farm and at population level.

**Supplementary Information:**

The online version contains supplementary material available at 10.1186/s12917-021-03003-8.

## Background

Scientific reports dealing with necrotic enteritis (NE) in turkeys are few and in most cases not comprehensive. *Clostridium perfringens* is considered the causative agent of most cases of NE in turkeys [1] as in broiler chickens. Because turkeys and chickens as well as their production systems differ in several ways, some aspects of NE in turkeys and chickens are also likely to differ. Specific studies on the epidemiology of NE in turkeys are therefore needed to help understand and prevent NE in this bird species.

Although NE in turkeys is rarely reported and discussed in the scientific literature, it is probably quite common [2]. In Norway NE in turkeys is the main leading cause of the use of therapeutic antibiotics in poultry. Due to the risk of development of resistance, it is desirable to reduce the use of therapeutic antibiotics as much as possible. Improved knowledge about, and management of, factors influencing the risk of NE is therefore of importance not only for improved turkey health, welfare and production performance but also for reduced risk of antibiotic resistance. Field data and challenge experiments have documented the role of *Eimeria spp.* as a risk factor for NE in broilers [3–5], and a predisposing role of *Eimeria spp*. in turkey NE has been suggested based on field data [2, 6] and experimental data [7].

This study aims to describe aspects of the epidemiology of NE in a population of commercial turkeys, including incidence, age distribution and the potential associations between disease occurrence and year, season, grow-out size and feed mill, controlling for the farm effect. The relationship between NE and coccidial oocyst counts is estimated in a subgroup of grow-outs. The prescription pattern for therapeutic antibiotics and the relationship between NE occurrence and production data are also reported.

## Results

### Bird age at NE detection

**Fig. 1:**
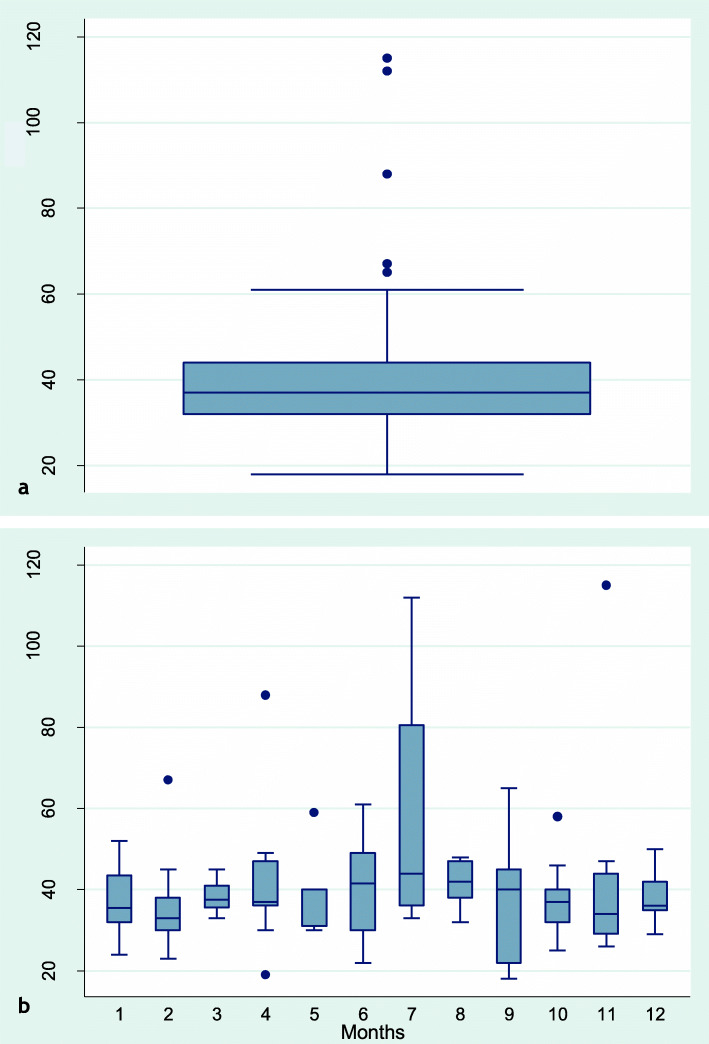
Distribution of age in days at detection of necrotic enteritis in 107 grow-outs of commercial slaughter turkeys. **a** All 107 grow-outs with age data. **b** Distributions of age at NE diagnosis per month (1 = January, 2 = February and so forth until 12 = December)

Age of grow-outs at NE detection was based on the first instance if NE was diagnosed several times during the same grow-out period. NE was diagnosed in 110 of 545 grow-outs and age at diagnosis was recorded in 107 of these. The median (min-max) age at NE detection was 37 (18–115) days. Mean age was 40 days. 80 % of all cases occurred at four to seven weeks of age (days 28–50). The age at NE detection tended to be higher in grow-outs hatched in June-August than during other parts of the year (Fig. 1b). The difference between grow-outs hatched in February as compared to July-August was significant (*p* = 0.03). The median age at NE detection among grow-outs hatched in February and July-August was 33 and 42 days, respectively.

### Incidence of necrotic enteritis

NE was reported in 110/545 grow-outs (20.2 %) started between August 2010 and October 2016. Among years with complete data (2010–2015) the percentage of grow-outs diagnosed with NE varied from a minimum of 10.6 in 2014 to a maximum of 28.6 in 2012.

Data on monthly incidences of NE (Table 1) indicate a distinct minimum incidence among grow-outs hatched during July-August and peak occurrences among grow-outs hatched during February-March and September-November. Monthly incidences show two steep increases (from grow-outs hatched in January to those hatched in February and from grow-outs hatched in August to those hatched in September) and a prolonged period of five months with continuously diminishing incidence (grow-outs hatched from February to July). Because most cases of NE appeared four to seven weeks after hatch, this means that the highest frequencies of NE were detected in March-April. In contrast, August-September was associated with the lowest NE level. The highest (28 %) and lowest (14 %) quarterly incidences were found in grow-outs hatched during the first and third quarters of the year, respectively. Thus, season appeared to be an important factor in NE incidence (Table 2).

**Table 1:** Monthly and quarterly incidences (%) of necrotic enteritis (NE)

Time component	Jan	Feb	Mar	Apr	May	June	July	Aug	Sept	Oct	Nov	Dec
Monthly %	22.6	36.8	26.1	23.3	17.1	14.0	9.3	10.6	22.0	22.5	22.4	17.3
Quarterly %	27.7			18.1			14.3			20.6		

Allocation of grow-out to month and quarter was based on the day of hatch. Data were collected from 545 grow-outs during years 2010–2016

**Table 2:** The final statistical model based on data from 545 turkey grow-outs started during 2010–2016

Variable	Variable level	Number of grow-outs	Odds ratio	*p*-value	95 % Confidence interval
Farm	Random effect	545	-	< 0.001	-
Season	January-March (reference)	137	1.00	-	-
	April-June	127	0.60	0.124	0.31–1.15
	July-September	140	0.41	0.007	0.21–0.79
	October-December	141	0.61	0.117	0.33–1.13
Grow-out size^a^	< 6000 (reference)	83	1.00	-	-
	6000 or more	462	2.78	0.052	0.94–7.47
Feed mill	1 (reference)	291	1.00	-	-
	2	163	1.32	0.456	0.63–2.77
	3	43	3.03	0.049	1.00-9.17
	4	27	0.78	0.734	0.19–3.23
	5^b^	21	1.43	0.600	0.37–5.51

Only variables associated with incidence of necrotic enteritis are displayed in the table

^a^Number of day-old turkeys housed on the same day on the same farm

^b^Grow-outs offered feeds from five different feed mills

### Necrotic enteritis and farm

A total of 32 farms from the most extensive study sample were represented by at least ten grow-outs each and a total of 442 grow-outs. NE was diagnosed in 20 % of these grow-outs, and 59 % of the NE cases were found in 25 % of these farms. NE incidence per farm varied between 0/25 grow-outs (< 4 %) and 10/17 grow-outs (59 %), suggesting a solid influence from farm.

### Necrotic enteritis and grow-out size

**Fig. 2:**
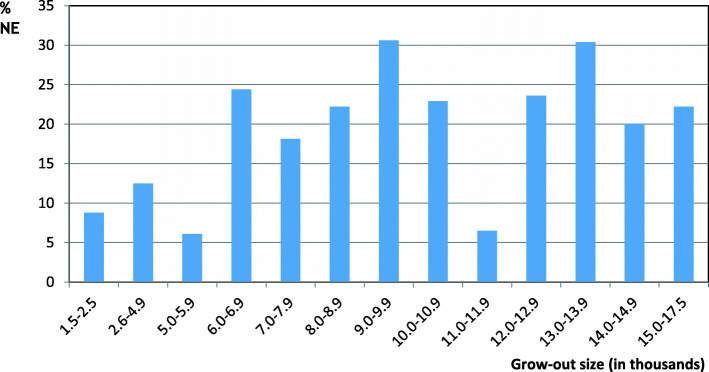
Percentage of grow-outs diagnosed with NE in 13 size categories based on numbers of day-old turkeys per grow-out. Numbers of day-old birds started (grow-out size) are indicated in thousands below each bar. The number of grow-outs per size category varied from 15 (14,000–14,999 day-olds) to 90 (6000–6999 day-olds)

The median grow-out size was 8200 birds (Table 3 ). Eight of ten grow-outs were started with 5000 to 13,000 day-old birds. The NE incidence among grow-outs smaller than 6000 birds appeared to be lower than the incidence among grow-outs of 6 000 birds or more (Fig. 2; Table 2).

**Table 3:** Grow-out size^a^ and age at slaughter of turkeys, based on data from 545 grow-outs

Population trait	Minimum	25 percentile	Median	Mean	75 percentile	Maximum
Grow-out size	1500	6700	8200	8687	11,000	17,028
Age females (days)	76	83	85	85	86	100
Age males (days)	113	128	132	131	135	144

^a^ Number of day-old poults

### Necrotic enteritis, feed mill and withdrawal of anticoccidial drugs

A total of nine feed mills provided feeds. NE was diagnosed in grow-outs supplied by all five mills providing feed to at least ten grow-outs. More than 91 % of the 545 grow-outs were provided with feeds from three different mills. One of these mills stood out with a higher percentage of NE grow-outs (46.5 % cases among 43 grow-outs) than the two others (16.2 and 21.5 % cases among 291 and 163 grow-outs, respectively). Data on time of anticoccidial drug withdrawal from the feed were available from 11 grow-outs supplied by these major feed mills. Anticoccidial drug was withdrawn at day 56 or later in seven grow-outs, between days 50–55 in two grow-outs and between days 42 and 49 in two grow-outs. These sparse data are supported by personal communications from the relevant feed companies, indicating that the majority of grow-outs were offered feed supplemented with anticoccidial drugs up to day 56 or more.

### The roles of variables potentially influencing necrotic enteritis incidence

Based on available data and preliminary examinations the following five categorical variables were formulated and selected for further analyses concerning association with NE incidence: Farm, Season (quarter at hatch), Year housed, Grow-out size (number of day-old birds per grow-out), and Feed mill. In order to examine the relationship between these variables and NE incidence in context, multivariable models with adjusted odds ratio estimates were built. A model including all five explanatory variables with Farm as a random effect indicated that Year housed was not significantly (*p* = 0.13 or higher for all years) associated with NE incidence. All other tested variables were recorded with p-values at 0.05 or lower in this model. Year housed was therefore removed from the model (Table 2). Removing any of the remaining variables did not improve the fit of the model. The final model was therefore.

$$\mathrm{Necrotic}\;\mathrm{enteritis}\;\mathrm{incidence}\;=\;\mathrm{Farm}\;+\;\mathrm{Season}\;+\;\mathrm{Grow}-\mathrm{out}\;\mathrm{size}\;+\;\mathrm{Feed}\;\mathrm{mill}$$

The logistic model does not give an exact measure of the variance, but based on the Pseudo R^2^ measure, 6.1 % of the variance of the data could be explained by the model. Random effect of farm was substantial (chi-square = 17.45, *p* < 0.001). Furthermore, the data indicate a clear impact of Season and more border-line impacts of Grow-out size and Feed mill (Table 2).

### Necrotic enteritis and faecal oocyst counts

**Fig. 3:**
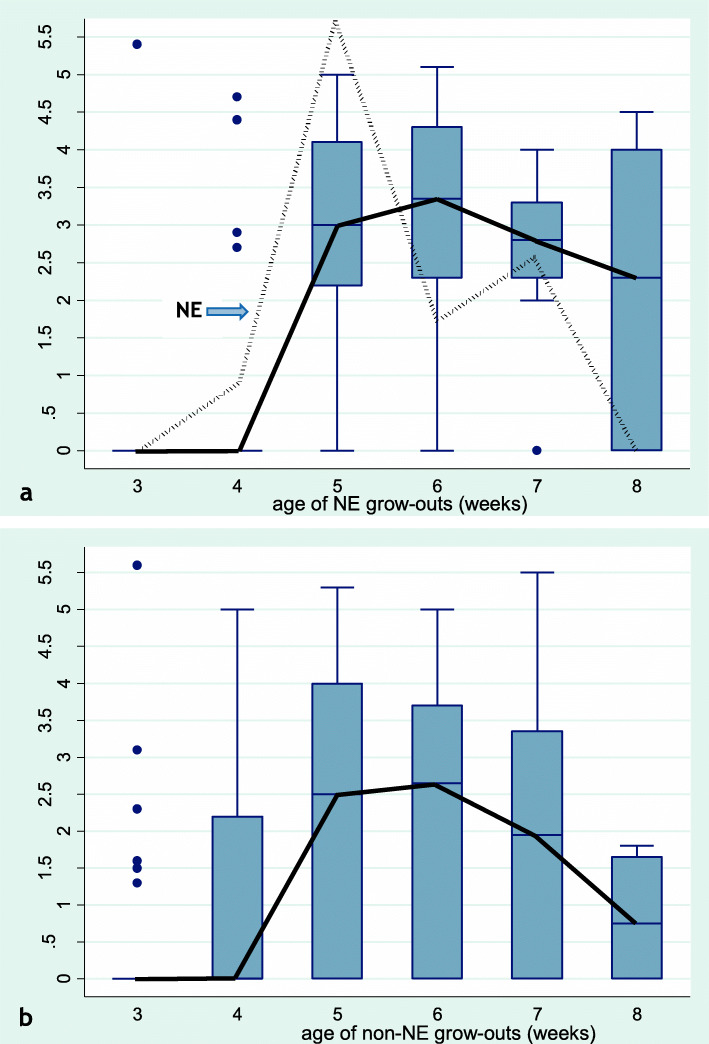
Box plots depicting the age dynamics of faecal oocyst counts (median counts: solid black line) per week of age. Y-axis indicates log_10_ oocysts per gram faeces (OPG). X-axis indicates age in weeks (3 to 8) of the examined turkey groups. **a** OPG of groups from grow-out diagnosed with necrotic enteritis (NE). NE occurrence is indicated as a dotted line (NE, arrow pointing at the line) in relative levels. The peak value at five weeks of age corresponds to detection of NE in 17.9 % of the grow-outs at that age. **b** OPG from grow-outs without NE diagnosis. The graphs are based on data from 39 grow-outs raised on 16 different farms

The relationship between faecal oocyst counts (OPG) counts and NE incidence was examined in data from 39 grow-outs. Figure 3a indicates that NE (dotted line) was detected during weeks 4 to 7 with the highest level of incidence during weeks 5 to 7. Median and mean age at detection of NE was 38 and 39 days, respectively. These figures are similar to the results from the study population of 545 grow-outs, suggesting that the subsample with OPG data was representative of the larger study sample regarding age pattern of NE occurrence.

Figure 3a also indicates a general concurrence in a rise of OPG counts and a rise of NE incidence, supporting a predisposing role of *Eimeria* in turkey NE. However, the picture is complex. Firstly, each grow-out consisted of at least two separate turkey groups; typically one group of females and one group of males. If NE was detected in only one turkey group, the whole grow-out was recorded as NE positive and treated with antibiotics. NE status was not recorded at the group level, hence we cannot analyse the direct relationship between OPG count and NE occurrence in each turkey group. OPG counts from grow-outs with NE in many cases represented a mix of samples from turkey groups with and without NE. However, if a predisposing role of high OPG counts is assumed, this would be expected to be reflected in a generally higher OPG level among grow-outs diagnosed with NE. A comparison of OPG counts of Fig. 3a (grow-outs diagnosed with NE) and Fig. 3b (grow-outs without an NE diagnosis) indicates a higher OPG level among grow-outs diagnosed with NE during the high-risk period weeks five to seven. This difference between grow-outs with and without NE was statistically significant (*p* = 0.03). The same trend (*p* = 0.07) was present during the whole age interval from three to eight weeks of age, although there appeared to be no difference in OPG counts between grow-outs with and without NE at three and four weeks of age.

### Necrotic enteritis and antibiotic therapy

A total of 127 of 545 grow-outs were treated at least once with therapeutic antibiotics. NE was the cause of treatment in 112 (88.2 %) of these cases. The two other most common causes of antibiotic treatment were gizzard inflammation and erysipelas.

Phenoxymethylpenicillin and amoxicillin were the two most commonly used therapeutic antibiotics (Table 4). There was an evident change in the prescription pattern during the study period, from the predominant use of amoxicillin to a predominance of phenoxymethyl-penicillin.

**Table 4:** Use of therapeutic antibiotics in Norwegian slaughter turkeys 2010–2016

Year	No. of treated grow-outs	Phenoxymethyl-penicillin	Amoxicillin	Other/Unknown
2010	4	-	100.0	-
2011	19	-	94.7	5.3
2012	25	16.0	76.0	8.0
2013	22	66.7	16.7	16.7
2014	10	80.0	20.0	-
2015	16	87.5	6.3	6.3
2016	16	93.8	-	6.3

Percentage of grow-outs treated due to necrotic enteritis, per type of antibiotic compound. Data from the Norwegian Meat and Poultry Research Centre Animalia

### Necrotic enteritis and foot pad scores

Foot pad scores were recorded at slaughter of 40 hen grow-outs. The median time interval between NE diagnosis and day of hen slaughter was 45.5 days. There was no significant difference (*p* = 0.52) in hen foot pad scores between grow-outs with (median score: 96) and without (median score: 94) a previous NE diagnosis. Most grow-outs were slaughtered more than three weeks after NE detection. Data from only two grow-outs that were slaughtered less than three weeks after NE detection (mean score: 120) were available.

### Necrotic enteritis and production performance

There was no association between a NE diagnosis and mean daily weight gain in female and male turkeys. The feed conversion ratio (FCR) was recorded for males and females taken together. Grow-outs diagnosed with NE tended to have less efficient feed conversion than grow-outs without this diagnosis (0.9 and 1.5 % differences for median and mean FCR, respectively). However, the association was not statistically significant (*p* = 0.12). A similar but slightly stronger association was found concerning profit per bird to the farmer (8.3 and 6.8 % differences for median and mean profit, respectively) with a p-value of 0.07 (Table 5).

**Table 5:** Production performance in grow-outs with and without a NE diagnosis

Production performance indicator	Not NE		NE		*P*-value
	mean (median)	n^a^	mean (median)	n^a^	
Weight gain females^b^	66.2 (65.7)	435	66.2 (66.0)	109	0.65
Weight gain males^c^	99.7 (98.7)	412	99.9 (98.8)	105	0.84
FCR (males + females)^d^	3.300 (3.300)	435	3.330 (3.345)	109	0.12
Profit (males + females)^e^	38.64 (37.50)	435	35.45 (34.95)	110	0.07

^a^Number of grow-outs

^b^Daily weight gain females (grams)

^c^Daily weight gain males (grams)

^d^Feed conversion ratio given as feed/kg slaughter weight

^e^Norwegian kroner (NOK) per bird

## Discussion

This study provides new and comprehensive data on the epidemiology of NE in commercial turkey meat production. NE was the most commonly diagnosed disease in this turkey population. The disease was deemed severe enough to be treated with antibiotics in the drinking water in 20.2 % of the 545 grow-outs that were started during 2010–2016. Previously published, solid population-based data on NE occurrence are absent (turkeys) or scarce (broilers) [8]. NE incidence seems to vary substantially over time in turkeys as well as in broilers. The incidence of clinical NE in our turkey population (quarterly incidence 14–28 %) appeared to be higher than mean levels in broilers reported before 1990 (5–9 %; these means include occurrence of epidemics), and more similar to peak levels reported during broilers epidemics (12–35 %) [8].

Median and mean age at the first instance of NE detection were 37 and 40 days, respectively. Eighty per cent of all cases appeared at four to seven weeks of age. Previous works [2, 9] have also reported distinct age intervals for the majority of NE cases in turkeys, with few cases appearing before six weeks of age and after 12 weeks of age. In our material the risk interval is earlier, which may be due to differences in production systems; e.g. housing, management, and feeding. Based on their field data, Droual et al. [2] suggest that there may be a resistance to NE in young poults, a finding that agrees with published experimental data [7, 10]. NE in commercial broilers is rarely found in birds younger than two weeks of age [11], and experimental NE in broilers is usually induced at about three weeks of age [5]. The age patterns of NE in turkeys and broilers thus appear to be similar in that NE is rarely found during the first few weeks of life.

Our data on the distribution of NE cases among farms and our final multivariable model (Table 3) indicates an apparent farm effect on NE incidence, suggesting that NE incidence might be substantially reduced using actions taken at farm level. The Farm variable comprises a multitude of factors (e.g. the quality of animal housing and other aspects of the physical environment, farm management quality, and biosecurity measures) whose relative importance cannot be evaluated based on the data available in this study. However, we do have data on two more specific aspects that also are related to the farm and its management; Grow-out size and Feed mill.

Feed mill was included (OR = 3.03, *p* = 0.05 for one feed mill, Table 3) in the multivariable model with Farm as a random effect. The mill with increased NE incidence supplied only six farms and 43 grow-outs with feeds, which may be seen as a cause for caution in emphasizing mill effect. However, the finding do suggest that some aspects of feed associated with mill (e.g. contamination with pathogenic *C. perfringens* strains, feed processing, feed structure, feed additives, ingredients and nutrients) might have influenced NE occurrence in these commercial turkey farms. This assumption is following previous findings in broiler chickens [8, 12, 13]. More work on specific factors related to nutrition and feeding of turkeys and their possible association with NE incidence is needed to determine the importance of such factors.

The binary variable Grow-out size assigned grow-outs to two categories: Those started with fewer than 6 000 birds and grow-outs started with at least 6 000 birds. Our data suggest an increasing trend in NE occurrence (OR = 2.8, *p* = 0.05, Table 3) with increased grow-out size. A possible reason for this trend might be that large grow-outs on average comprised higher numbers of turkey groups. Even with the same risk of NE per turkey group in small and large grow-outs, the risk of NE per grow-out would therefore be higher among large grow-outs. An alternative or supplementary explanation could be that a large grow-out is a risk factor *per se*. If this is the case, the current trend of increasing farm and grow-out size demands even more focus on measures against NE.

In our study of *Eimeria* oocyst counts (OPG) from 39 grow-outs sampled during 2015–2017 (including 36 of the grow-outs from the larger study population) we found that OPG counts and NE incidence peaked more or less concurrently at around five to six weeks of age (Fig. 3). Furthermore, the increase in OPG values was most marked among grow-outs diagnosed with NE. These findings suggest that subclinical coccidiosis may have been a predisposing factor contributing to the peak occurrence of NE at around five to seven weeks of age. This conclusion based on data from commercial farms is supported by experimental results indicating that coccidial challenge at five (but not at three) weeks of age combined with *Clostridium perfringens* challenge is an effective means of reproducing severe intestinal lesions in turkeys [7]. The role of *Eimeria* as a predisposing factor for NE has also been demonstrated in broiler experiments [5], although different *Eimeria* species appear to differ in their ability to induce NE mortality [4]. Early withdrawal of in-feed anticoccidial drugs might have contributed to increased OPG counts and NE in some cases. However, based on available information about the timing of withdrawals (not prior do day 42) and peak occurrence of NE (before day 42), anticoccidial drug withdrawal seems an unlikely main driver of NE appearance. In many cases neither high OPG counts nor NE was prevented by in-feed anticoccidial drugs.

Season of the year affected NE occurrence in two ways; age at appearance (Fig. 1) and incidence (Table 2). Median age at NE detection was at or above 40 days in grow-outs hatched during months June to September (i.e. in grow-outs with NE outbreaks in July to October), and below 40 days during all other months. NE appeared particularly early in grow-outs hatched in February. Grow-outs hatched in February also had the highest incidence of NE (36.8 %), whereas grow-outs hatched in July were recorded with the lowest incidence (9.3 %). These findings indicate that season-dependent factors influence age pattern and occurrence of NE, with peak NE incidence during March-April (four to seven weeks after hatch in February). Climate is a possible element in this context, by influencing the environment in the turkey house, and consequently the conditions for the proliferation of pathogens. Moist litter [14] due to condensation caused by cold weather and restricted ventilation may promote *Eimeria* proliferation and subclinical coccidiosis, and thus predispose the turkeys to NE [2, 6, 7]. However, cold weather alone cannot explain the peak occurrence in March-April, because January and February usually are colder months at the locations of these farms (Table 6). A possible explanation might be that heating of the coldest inlet air in January and February leads to low relative humidity, which delays oocyst sporulation. A combination of changing weather conditions and sub-optimal management of in-door climate may explain the peak occurrence of NE in March and April.

**Table. 6:** Location of turkey farms, and temperature (°C) and precipitation (mm) data

Location	Climate variable^a^	Jan-Mar	Apr-June	July-Sep	Oct-Dec
Hedmarken (Hamar)	Temperature	-2 to -1, -24, 10	2 to 16, -5, 31	8 to 17, 1, 28	-6 to 8, -9, 16
	Precipitation	15–22	28–56	56–68	26–49
Østfold (Rakkestad)	Temperature	-3 to 2, -23, 13	2 to 15, -6, 31	8 to 16, 0, 29	-3 to 8, -8, 17
	Precipitation	49–64	49–76	82–102	85–104
Vestfold (Tjølling)	Temperature	-1 to 4, -17, 14	4 to 16, -4, 29	10 to 17, 2, 27	0 to 10, -5, 16
	Precipitation	49–55	55–64	79–117	84–132

^a^Temperature: Lower and upper normal temperatures per quarter (upper row in each cell), and minimum and maximum temperature during the 13 month period including May 2020 to May 2021 (lower row in each cell). The normal temperature is based on data from 1991–2020 (https://www.met.no/vaer-og-klima/ny-normal-i-klimaforskningen). Precipitation: Lower and upper normal amount (mm of water) of monthly precipitation per quarter. Data based on records from three representative locations (https://www.yr.no/nb/historikk/)

Furthermore, it is clear from the data (Fig. 3) that relatively high OPG counts in some turkey groups were not always associated with an NE diagnosis. NE grow-outs comprised only one third (21/64) of the turkey groups with OPG levels at or above 10 000, which suggests that other factors were also significant predictors of an NE diagnosis.

The potential role of haemorrhagic enteritis (caused by turkey adenovirus A species in genus *Siadenovirus*) as a predisposing factor for NE was discussed by Droual et al. [2]. They argued that because this virus is prevalent in turkeys, and the disease is associated with immunosuppression and occurs during the same age interval as NE, haemorrhagic enteritis may increase the likelihood that NE will occur. Our study was not designed to investigate the role of haemorrhagic enteritis, but it is noteworthy that this disease was diagnosed six days before an NE diagnosis in one of the grow-outs with NE that were examined for OPG counts.

The use of therapeutic antibiotics was, to a large extent, determined by NE incidence. The observed change in prescription pattern from amoxicillin to phenoxymethyl-penicillin was the result of a deliberate choice that took place because phenoxymethyl-penicillin has a more narrow spectrum of activity, and therefore is more targeted against *Clostridium perfringens* causing NE, and less likely to induce antibiotic resistance in other pathogens.

NE did not influence the daily weight gain of female or male turkeys. The values of this variable were based on achieved growth from the day of hatch until slaughter at about 85 (females) or 132 (males) days of age. This lack of influence of NE on weight gain does not exclude the possibility that NE temporarily reduced weight gain. Compensatory growth may have taken place during the weeks between NE occurrence and slaughter (48 days difference between median age at NE and median age at hen slaughter). Furthermore, the estimated sex-specific association between NE and weight gain could have been weakened by the likely fact that some grow-outs diagnosed with NE were affected by NE in one sex only. The same considerations apply to the apparent lack of impact of NE on foot pad scores. Published experimental results [15] suggest that diarrhoea caused by coccidial infection can lead to poor litter quality, and hence, increased severity of foot pad dermatitis in turkeys. Because coccidial infection was a likely predisposing factor for NE in our observational study, NE may also have been associated with higher foot pad scores during the first few weeks following disease outbreaks, as suggested by the higher scores of the two only grow-outs that were slaughtered less than three weeks after NE detection. However, most grow-outs were examined for foot pad lesions six to seven weeks after NE diagnosis, which may have provided time for re-establishment of a satisfactory litter quality and healing of foot pad lesions.

In this study the feed conversion ratio was estimated based on merged data from males and females in the same grow-out, since most farmers do not have separate feeding systems for males and females. Because most NE cases appeared around 5 to 6 weeks of age, the impact of NE on accumulated feed conversion ratio was likely to be more modest in males than in females. Furthermore, feed conversion results from males were given more weight than the results from females, because the males were substantially larger and had consumed much more feed than the females. These circumstances may have contributed to the lack of significant (*p* = 0.12) impact of NE on feed conversion ratio. It is however noteworthy that grow-outs diagnosed with NE were estimated with about 1 % poorer feed conversion than grow-outs without NE. The same considerations apply to profit margin per bird. In this case our data indicate a lower p-value (0.07) and higher estimates (seven to 8 %) for a negative impact of NE.

## Conclusions

The incidence of necrotic enteritis in turkeys was strongly influenced by season and farm. The strong farm effect underlines the potential importance of environmental factors and/or management factors in the epidemiology of this disease. Our data suggest that subclinical coccidiosis was an important predisposing factor for NE in the examined turkey population. Although not investigated in this study, variable severity of coccidiosis might also have been associated with farm management and season. The potential roles of diet and grow-out size in the epidemiology of necrotic enteritis in turkeys deserve further studies.

## Methods

### Study design and populations

This observational study comprises two study populations of B.U.T. 10 turkeys.

The largest study population consists of all grow-outs (545 grow-outs from 57 commercial turkey farms) that were started in south-eastern Norway during the period August 2010 to October 2016. All farms were located in the three regions (Hedmarken, Vestfold, Østfold) with the majority of turkey farms in the country (as from 2018 the only regions). Data on outdoor temperatures and precipitation in these three regions are displayed in Table 6.

A grow-out was defined as the entire group of day-old birds that were housed on the same day on the same farm. Data from these grow-outs were collected routinely by the company (Nortura SA, Norway) slaughtering turkeys from these farms, and made accessible for this study. All grow-outs were started with female turkeys, and 95.2 % of the grow-outs also included males. Females and males were raised separately as from the day of hatch until slaughter. All birds were kept on litter floor, and offered free access to feed and water. Some farms divided male and female groups into more sub-groups during the first eight weeks of rearing. This means that whereas some sub-groups were kept on partly the same litter floor during the whole grow-out period, others were moved to other rooms or houses with fresh litter at some time point after initial housing. All grow-outs were started with fresh litter material. Females were usually slaughtered at about 12 weeks of age, and males were mostly slaughtered at about 19 weeks of age. The percentage of grow-outs started per month varied between a minimum of 7.0 of all started grow-outs in February and a maximum of 9.7 in January.

A smaller study population of 39 grow-outs started during 2015–2017 at 16 commercial turkey farms were used to study the relationship between NE occurrence and levels of *Eimeria* oocyst counts per gram faeces (OPG) in three to eight weeks old birds. Based on historical data on NE occurrence, farms were selected in order to ensure a sample of grow-outs that was representative of the larger study population, which was confirmed by comparing data on NE frequency and average age of turkeys at NE appearance from the two study populations. Data from 14 of the farms (36 grow-outs) in the smaller study population constituted a subset of the study with 545 grow-outs. Data on withdrawal time of anticoccidial drugs were collected from 12 of the grow-outs in the smaller study population.

All grow-outs in both study populations were started with in-feed anticoccidial drugs, mainly monensin and to a minor extent lasalocid. These anticoccidials were used continuously until withdrawal. Only one type of anticoccidial compound was used in each grow-out; no shuttle or systematic rotation programs were used. Withdrawal took place at six to nine weeks of age, in most cases at about eight weeks of age. No anticoccidial vaccines or antibiotic growth promoters were used.

### Incidence of necrotic enteritis in the largest study population

NE was diagnosed by Nortura’s field veterinarians, based on gross lesions. Characteristic findings include small intestinal pseudomembranes with a mucoid appearance, often accompanied by gas-filled intestines with watery contents. NE was recorded if the outbreak was deemed severe enough to require treatment with therapeutic antibiotics. NE incidence was defined as the percentage of all 545 grow-outs diagnosed with at least one recorded outbreak during the whole study period or during specific time components (yearly, quarterly or monthly time intervals). Each grow-out was allocated to time interval based on the date of hatch.

### Sampling of faeces and counts of oocyst per gram (OPG) in the smaller study population

OPG counts were estimated and recorded in 39 grow-outs from 16 farms. NE incidence was calculated as the number of detections of NE that led to antibiotic treatment up to and including eight weeks of turkey age. Whereas OPG counts were recorded at the turkey group level (at least two groups per grow-out) on several (mainly three to five) occasions between three and eight weeks of age, NE incidence was recorded at the grow-out level. Pooled samples of fresh faeces were collected from five evenly distributed areas within the floor space shared by each turkey group. Litter was included in the sampling material only to the extent that it was inseparable from wet faeces. Each of the samples pooled per turkey group and sampling day were mixed thoroughly before being examined.

The levels of oocysts per gram faeces (OPG) were determined using a modified McMaster method for parasite egg counting. This method is based on published literature [16]. Briefly, the protocol is based on dilution of faeces and subsequent flotation of oocysts before counting; 30 g of thoroughly mixed pooled sample, 420 ml tap water, mixed in a blender and sieved through a sieve with 250 μm mesh size, centrifugation for 3 min at 3000 rotations/minute, removal of the supernatant from the oocyst-containing sediment, mixing of oocysts in saturated NaCl with a volume corresponding to the supernatant, and examination of 1,0 ml this mixture using a Whitlock Universal counting chamber. This method has a theoretical lower sensitivity of 15 OPG.

### Foot pad scoring

Foot pads from 100 feet per grow-out were scored for lesions from 0 to 2 (0: no lesions, 1: moderate lesions, 2: severe lesions) at the slaughter of 260 grow-outs. Sum of foot pad score per grow-out was calculated based on the following formula:

$${\mathit(number\mathit\;of\mathit\;feet\mathit\;with\mathit\;score\mathit\;\mathit0\mathit\;\times\mathit\;\mathit0\mathit)}\mathit\;+\mathit\;{\mathit(number\mathit\;of\mathit\;feet\mathit\;with\mathit\;score\mathit\;\mathit1\mathit\;\times\mathit\;\mathit1\mathit)}\mathit\;+\mathit\;{\mathit(number\mathit\;of\mathit\;feet\mathit\;with\mathit\;score\mathit\;\mathit2\mathit\;\times\mathit\;\mathit2\mathit)}$$

Range of sum of foot pad scores was therefore 0 to 200. Foot pads were scored at slaughter; i.e. at 68–108 days of age for females and at about 19 weeks of age for males. Because 90 % of all NE cases appeared in grow-outs below 51 days of age, only foot pad scores in females (scored 18–51 days after NE detection) were examined.

### Production performance

Daily weight gain was calculated per sex and grow-out, based on mean carcass weight at slaughter. The feed conversion ratio was calculated per grow-out (including both females and males), based on accumulated feed uptake at slaughter and carcass weight at slaughter. Calculation of profit per bird to the farmer was based on costs of day-old poults and feed, and payment per approved carcass.

### Statistical analysis

All data were collated in Excel spread-sheets.

Raw data on explanatory variables in the largest study population were provided by Nortura SA in an Excel spreadsheet format, and were further analysed in Excel before being imported into Stata 14.2 or Stata 16.1 for statistical analysis. The relationship between each variable and NE occurrence was explored and described in text, tables, and figures. The unit of concern in these analyses was grow-out. The outcome was binary (NE yes/no). Explanatory variables were categorical (Farm, Year housed, Season, Feed mill) or were made binary (Grow-out size: number of day-old turkeys per grow-out). A multilevel mixed-effects logistic regression model was built using the *melogit* procedure in Stata 16.1 with NE as the outcome, and Farm, Season, Feed mill, and Grow-out size as predictors. Farm was included as a random effect to adjust for the repeated structure of data. A backward selection approach was used to build the final model.

The Kruskal-Wallis rank sum test (*kwallis* procedure in Stata 14.2) was used to compare performances of grow-outs (*N* = 545) with and without an NE diagnosis (Table 5), to compare log_10_ OPG counts (*N* = 39) and foot pad scores (*N* = 545) in grow-outs with and without NE, and to compare seasonal differences in age at NE occurrence among grow-outs diagnosed with NE (*N* = 107).

## Supplementary Information

Below is the link to the electronic supplementary material.

**Additional file 1.** Data supporting conclusions regarding factors associated with necrotic enteritis incidence [Data 545 grow-outs].

**Additional file 2.** Data supporting conclusions regarding an association between faecal oocyst counts and necrotic enteritis incidence [Data OPG].

## Data Availability

Two datasets that support the findings of this article are included as additional files.

## Abbreviations

FCR: Feed conversion ratio (kg feed/kg carcass weight)
NE: Necrotic enteritis
OPG: Number of *Eimeria* oocysts per gram faeces
